# Metabolomics analysis reveals a modified amino acid metabolism that correlates with altered oxygen homeostasis in COVID-19 patients

**DOI:** 10.1038/s41598-021-85788-0

**Published:** 2021-03-18

**Authors:** José C. Páez-Franco, Jiram Torres-Ruiz, Víctor A. Sosa-Hernández, Rodrigo Cervantes-Díaz, Sandra Romero-Ramírez, Alfredo Pérez-Fragoso, David E. Meza-Sánchez, Juan Manuel Germán-Acacio, José L. Maravillas-Montero, Nancy R. Mejía-Domínguez, Alfredo Ponce-de-León, Alfredo Ulloa-Aguirre, Diana Gómez-Martín, Luis Llorente

**Affiliations:** 1 Red de Apoyo a la Investigación, Universidad Nacional Autónoma de México e Instituto Nacional de Ciencias Médicas y Nutrición Salvador Zubirán, Mexico City, Mexico; 2 Emergency Medicine Department, Instituto Nacional de Ciencias Médicas y Nutrición Salvador Zubirán, Mexico City, Mexico; 3 Departament of Immunology and Rheumatology, Instituto Nacional de Ciencias Médicas y Nutrición Salvador Zubirán, Vasco de Quiroga 15, Sección XVI, C.P. 14000 Tlalpan, Mexico City, Mexico; 4 Departamento de Biomedicina Molecular, Centro de Investigación y de Estudios Avanzados del Instituto Politécnico Nacional, Mexico City, Mexico; 5 Facultad de Medicina, Universidad Nacional Autónoma de México, Mexico City, Mexico; 6 Department of Infectology and Microbiology, Instituto Nacional de Ciencias Médicas y Nutrición Salvador Zubirán, Mexico City, Mexico

**Keywords:** Metabolomics, Viral infection, Hypoxia

## Abstract

We identified the main changes in serum metabolites associated with severe (n = 46) and mild (n = 19) COVID-19 patients by gas chromatography coupled to mass spectrometry. The modified metabolic profiles were associated to an altered amino acid catabolism in hypoxic conditions. Noteworthy, three α-hydroxyl acids of amino acid origin increased with disease severity and correlated with altered oxygen saturation levels and clinical markers of lung damage. We hypothesize that the enzymatic conversion of α-keto-acids to α- hydroxyl-acids helps to maintain NAD recycling in patients with altered oxygen levels, highlighting the potential relevance of amino acid supplementation during SARS-CoV-2 infection.

## Introduction

COVID-19 is an emerging infection caused by the severe acute respiratory syndrome (SARS)-CoV-2 virus, whose main clinical features are fever, fatigue, cough, lymphopenia and, in severe cases, pneumonia with SARS, which if uncontrolled leads to multi-organic failure and dead^1^. In severe cases, hypoxia seems to play a distinct role in the adverse clinical outcomes of COVID-19 patients. Therefore, several clinical and pharmacological approaches for restoring oxygen levels to viable parameters are currently under evaluation^2^.

The physiological changes related to the presence of hypoxia that occur as an attempt to normalize oxygen levels include increase in respiratory rate, vasodilation, and vascularization as mechanisms to fulfill energetic requirements until normoxia is attained^3^. At the molecular level, hypoxia inhibits oxidative phosphorylation in mitochondria, which is the main source of adenosine triphosphate (ATP) for critical and energetic expensive processes such as ionic equilibrium and protein synthesis^3^.

Hypoxia inducible factor 1-α (HIF 1-α) is the master regulator of oxygen homeostasis in mammals and has crucial roles on metabolism through up-regulating genes involved in the glycolytic pathway during hypoxic conditions^4^. The activity of this transcription factor favors anaerobic glycolysis over mitochondrial respiration as a source of ATP when environmental O_2_ falls^4^. Despite that anaerobic glycolysis produces only 2 ATP molecules per molecule of glucose, this metabolic shift is accompanied by an increased glucose influx as a compensatory mechanism to reach high levels of ATP^4^. However, a sustained glycolytic metabolism may potentially lead to reduced nicotinamide dinucleotide (NAD) pools, which must be preserved to keep the cytosolic synthesis of ATP during hypoxia^5^. Although pyruvate is considered the final electron acceptor in this pathway, oxidation of this α keto-acid (α-KA) by lactate dehydrogenase is not the only way to replenish NAD levels in hypoxia. Other α-keto-acid oxidases, such as malate dehydrogenase, are able to replenish the cytosolic NAD pools in hypoxic environments^6^.

In the present study, our metabolomics analysis disclosed dysregulation of pathways linked to energy production (Krebs cycle, Warburg effect) and amino acid catabolism [BCAA (branched chain amino acids)], threonine, glutamine and glutamate) in COVID-19 patients. An increment of three different α hydroxyl acids (α-HA) linked to valine and threonine catabolism in infected patients was prominent, leading to the proposal that in hypoxic patients the α-KA derived from these amino acids become the final electron acceptors through the activity of their corresponding α-keto-acid oxidases. Accordingly, the increment of serum glutamate in COVID-19 patients could be related with an increased transaminase activity, an enzymatic process that generates α-KA as reaction products^5^.

Our results are in line with other COVID-19 metabolomics analyses, with emphasis on the relevance of amino acid catabolism during hypoxic conditions^7–9^. Interestingly, these metabolic signatures may have implications on the appearance of adverse effects due to SARS-CoV-2 infection, such as diabetes and neurological disabilities.

## Results

### Demographic and clinical features of control donors and patients with COVID-19

Sixty-five COVID-19 patients were included, 19 with mild disease and 46 with severe disease. In addition, 27 subjects with a negative PCR test for SARS-CoV-2 infection were included. The main clinical characteristics and demographic features of the patients and controls are shown in Table 1.

**Table 1:** Demographic, clinical, and laboratory parameters of the participants.

Parameter	Control donors	Mild COVID–19	Severe COVID-19	P-value
**Demographic features**				
Sex	F:17–62.9%	F:9–47.4%	F:17–36.9%	0.098
F: Female	M:10– 37.1%	M:11–52.6%	M:29–63.0%	
M: Male	Total: 27	Total: 19	Total: 46	
Age (Average-SD)	35.4 ± 8.8	40.7 ± 10.7	49.6 ± 13.6	2.658 × 10^–5^
BMI (Average SD)	26.9 ± 4.3	26.9 ± 3.4	27.8 ± 3.8	0.011
**Comorbidities**				
Overweight	11/27—40.7%	3/19—15%	15/46—32%	0.195
Obesity	5/27—18.5%	3/19—15%	20/46—42.6%	0.014
DM2	2/27– 7.4%	2/19—10%	12/46—25.5%	0.085
Hypertension	1/27—3.7%	1/19—5%	17/46—36.2%	5.697 × 10^–4^
Cardiopathy	0–0%	0—0%	5/46—10.6%	0.1146
**Vital signs**				
Temp (ºC)	–	37.4 ± 0.7	37.2 ± 0.8	0.095
Hearth rate (bpm)	–	101.3 ± 19.3	103.6 ± 17.0	0.680
Respiratory rate (bpm)	–	20.1 ± 3.8	25.2 ± 6.5	0.002
Mean arterial pressure (mmHg)	–	95.1 ± 11.6	97.6 ± 14.9	0.982
SO2 (%)	–	94.8 ± 1.4	86.5 ± 9.2	4.889 × 10^–8^
**Symptoms**				
Cough	–	15/19—75%	38/46—80.9%	0.734
Headache	–	15/19—75%	24/46—51.1%	0.055
Fever	–	16/19—80%	42/46—89.4%	0.407
Dyspnea	–	5/19—25%	33/46—70.2%	0.001
Arthralgia	–	11/19—55%	26/46—55.3%	0.999
Myalgia	–	11/19—55%	31/46—66%	0.570
Sore Throat	–	5/19—25%	15/46—31.9%	0.770
Nausea	–	1/19—5%	4/46—8.5%	0.999
Diarrhea	–	2/19—10%	10/46—21.3%	0.483
Fatigue	–	9/19—45%	20/46—42.5%	0.790
**Arterial blood gases**				
FiO2 (%)	–	–	27.9 ± 12.6	
pH art	–	–	7.33 ± 0.2	
Arterial PO2 (mmHg)	–	–	83.7 ± 43.2	
Arterial PCO2 (mmHg)	–	–	28.9 ± 3.7	
HCO3 (mmol/L)	–	–	21.4 ± 2.8	
Lactate (mmol/L)	–	–	1.67 ± 0.9	
PAFI	–	–	265.7 ± 104.3	
Anion gap (mmol/L)	–	–	14.3 ± 2.4	
**Laboratory tests**				
Glucose (mg/dL)	–	–	134.04 ± 76.2	
CRP (mg/dL)	–	–	13.3 ± 8.4	
Ferritin (ng/mL)	–	–	555.3 ± 408.2	
DHL (U/L)	–	–	373.07 ± 162.8	
ALT (U/L)	–	–	39.06 ± 36.5	
AST (U/L)	–	–	41.6 ± 29.7	
Creatinine (mg/dL)	–	–	0.97 ± 0.3	
Albumin (g/dL)	–	–	3.99 ± 0.8	

*P*-value for Kruskal–Wallis, U, cχ^2^ or Fisher exact test.

Using an untargeted metabolomics approach, we determined 46 different metabolites ([< 30% relative standard deviation (RSD) in quality control (QC) samples] in serum samples from patients and controls. The main characteristics of these compounds are shown in Table S1. The method showed high reproducibility as observed by principal component analysis (PCA) of QC samples, which forms a well-defined compact cluster (Figure S1).

**Figure 1:**
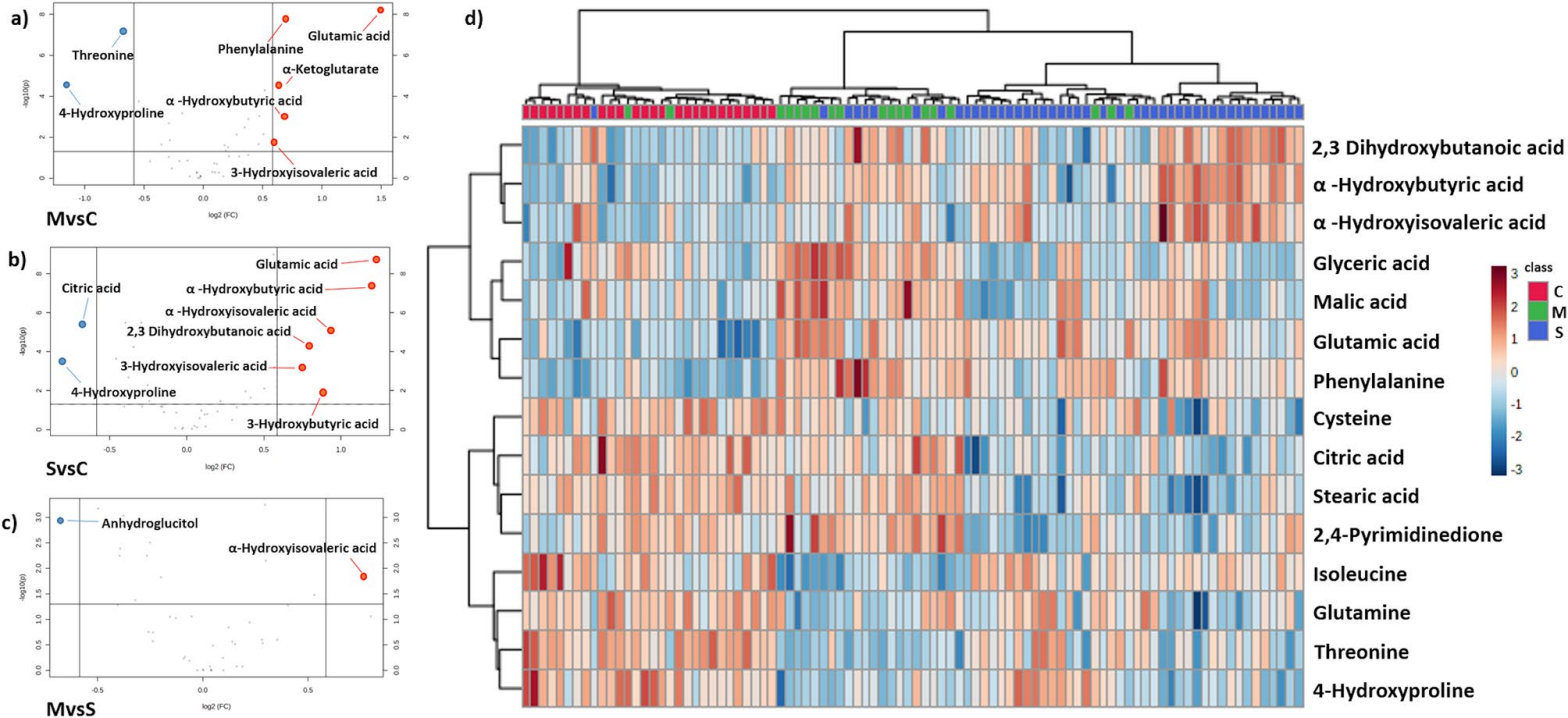
Changes in serum metabolites of controls *vs* COVID-19 patients. Volcano plots of metabolites that increase (red) or decrease (blue) in (**a**) mild COVID-19 versus controls, (**b**) severe COVID-19 vs controls and (**c**) severe versus mild COVID-19. Cut off fold change > 0.5, *p* value < 0.05. (**d**) Heatmap visualization and clustering analysis of differential metabolites among controls (C), mild (M) and severe (S) COVID-19 patients. Only the 15 metabolites with the lowest *p* values (by ANOVA) are shown.

We performed volcano plots analysis to identify those metabolites with the highest changes between the groups (Fold change > 0.5 and p.-adjusted values < 0.05, Fig. 1). When compared to control, we detected lower levels of threonine and citrate in COVID-19 patients. Inversely, α-Ketoglutarate, phenylalanine, glutamic acid and several intermediaries of amino acid catabolism (3-Hydroxyisovaleric, 3-Hydroxybutyric acid, α-Hydroxybutyric acid, α-Hydroxyisovaleric, 2,3-Dihydroxybutanoic acid) increase with disease severity. Only α-Hydroxyisovaleric acid (a metabolite of valine degradation pathway) increases between mild and severe patients (Table 2).

**Table 2:** Comparison of metabolites with fold changes > 0.5 according to disease severity.

Combination	Metabolite	FC	log2(FC)	p-.adjusted	Kruskal–Wallis CvsMvsS p.-adjusted
M/C	Glutamic acid	2.8108	1.491	5.89 × 10^–9^	M–C; S–C 2.21E−10–4.96E−09
	Phenylalanine	1.616	0.69239	1.73 × 10^–8^	M–C; S–C 1.38E−10–4.96E−09
	α-Hydroxybutyric acid	1.5999	0.67798	9.26 × 10^–4^	M–C; S–C; S–M 5.73E−09–8.59E−08
	α-Ketoglutarate	1.5639	0.64511	3.04 × 10^–5^	M–C; S–C; M–S 0.00021638–0.00060858
	3-Hydroxyisovaleric acid	1.5081	0.59277	0.02247	M–C; S–C 0.00094552–0.0018499
	Threonine	0.62777	− 0.6717	6.68 × 10^–8^	C–M; C–S 5.73E−09–8.59E−08
S/C	Glutamic acid	2.3519	1.2338	1.45 × 10^–9^	M–C; S–C 2.21E−10–4.96E−09
	α-Hydroxybutyric acid	2.3094	1.2075	4.04 × 10^–8^	M–C; S–C; S–M 5.73E−09–8.59E−08
	α-Hydroxyisovaleric acid	1.9297	0.94837	7.55 × 10^–6^	S–C; S–M 3.04E−06–1.71E−05
	3-Hydroxybutiric acid	1.8519	0.88901	0.012145	S–C; S–M 0.008355–0.012532
	2,3-Dihydroxybutanoic acid	1.7324	0.79276	5.73 × 10^–5^	M–C; S–C 5.43E−05–0.00024421
	3-Hydroxyisovaleric acid	1.6932	0.75971	5.46 × 10^–4^	M–C; S–C 0.00094552–0.0018499
	Citric acid	0.62276	− 0.68326	3.21 × 10^–6^	C–S; M–S 9.24E−07–7.07E−06
	4-Hydroxyproline	0.57272	− 0.80409	2.77 × 10^–4^	C–M; C–S 1.09E−05–5.47E−05
M/S	α-Hydroxyisovaleric acid	1.7032	0.76821	0.014705	S–C; S–M 3.04E−06–1.71E−05
	Anhydroglucitol	0.62921	− 0.66838	0.0011881	M–C; M–S 0.0004461–0.00095594

Fold-change was calculated using Metaboanalyst 5.0. *p* values were calculated using the Mann–Whitney test for MvsS and Kruskal Wallis test for multiple comparisons (CvsMvsS). Comparisons are included as follows: mild COVID-19 versus controls (MvsC); severe COVID-19 versus controls (SvsC) and severe versus mild COVID-19 (SvsM).

To obtain an overview of the main changes in our metabolomics data, we performed a heatmap and hierarchical clustering analysis using the 15 metabolites with the lowest p value (ANOVA *p* < 0.05) (Fig. 1). With this approach, we detected reduced levels of several amino acids (cysteine, isoleucine, glutamine, and threonine) and lower levels of glyceric and citric acid in COVID-19 patients. Conversely, increased levels of three different α-HA (α-hydroxyisovaleric acid, α-hydroxybutyric and 2,3 dihydroxybutanoic acid and malic acid) and two amino acids (glutamic acid and phenylalanine) were identified in infected patients. However, this analysis is not able to generate a perfect clustering between the analyzed groups and to discriminate for disease severity.

**Figure 2:**
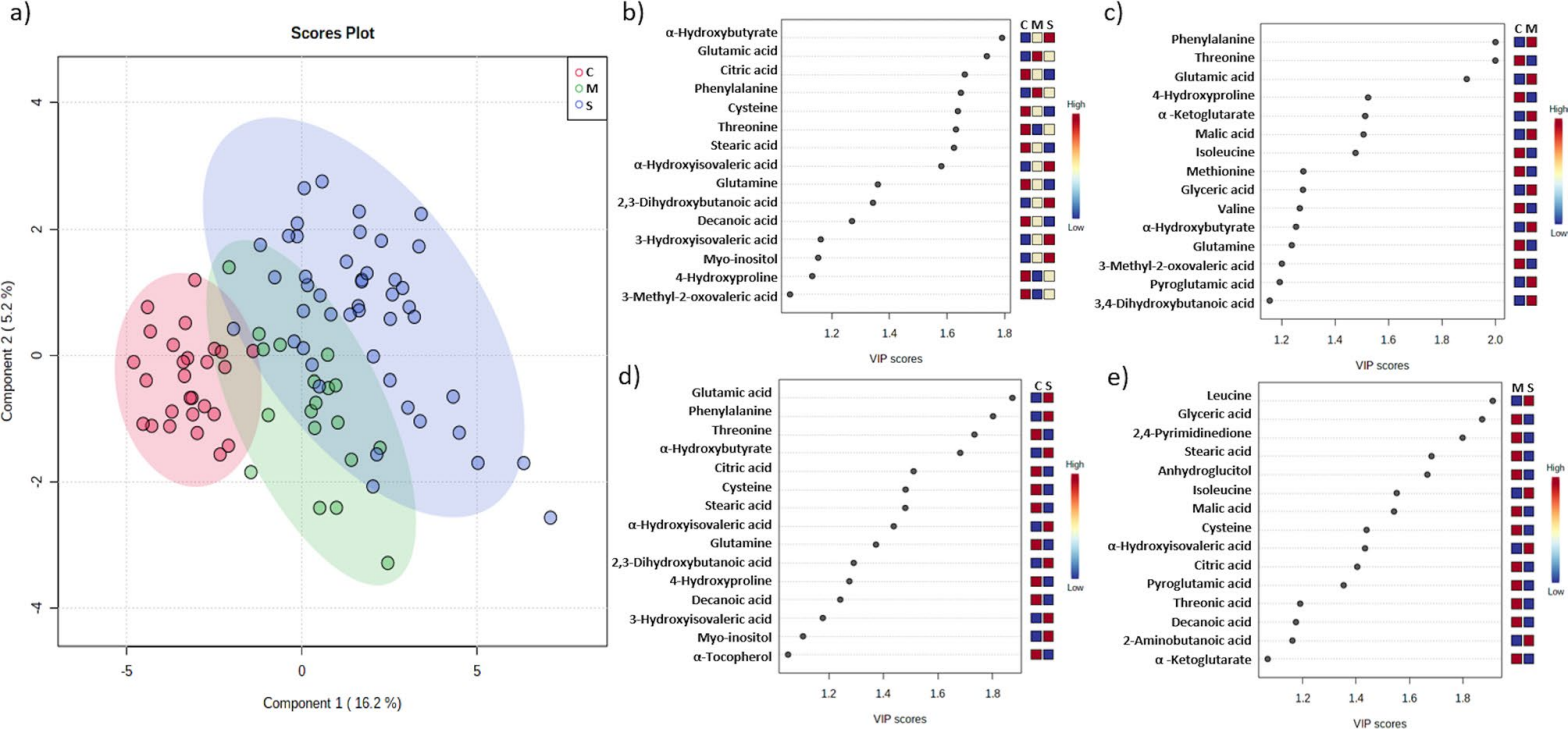
Partial least squares-discriminate analysis (PLS-DA) plot of differential metabolites from control and COVID-19 patients and their corresponding VIP values. (**a**) PLSDA score plots for control (H-red), mild (M-green) and severe (S-blue) patients .VIP schematic scores of PLS-DA analysis for CvsMvsS (**b**), CvsM (**c**), CvsS (**d**), and MvsS (**e**) groups.

To further categorize the patients according to the metabolic profile, we performed a supervised multivariate partial least squares-discriminate analysis (PLS-DA). To assess the robustness of the model classification, cross validation and permutation analysis were performed for the following comparisons: Control versus severe COVID-19 (CvsS), control versus mild COVID-19 (CvsM), mild COVID-19 versus severe COVID-19 (MvsS), and control versus mild COVID-19 versus severe COVID-19 (CvsMvsS), obtaining good prediction parameters except for the MvsS groups, indicating a less metabolic variation in this set of subjects (Table 3). The Variable Importance to the Projection (VIP) metabolites relevant to group each cluster are depicted in Fig. 2; as shown, VIP values belong to amino acid, α-HA, fatty acids, and Krebs cycle intermediaries.

**Table 3:** Predictability values of partial least squares-discriminate analysis (PLS-DA).

Measure	C *vs* M	C *vs* S	M *vs* S	C *vs* M *vs* S
R22	0.836	0.845	0.568	0.797
Q22	0.691	0.723	0.262	0.667
Accuracy	0.957	0.973	0.831	0.826

C versus M, Control versus Mild; C versus S, Control versus Severe; M versus S, Mild versus Severe; and C versus M versus S, Control versus Mild versus Severe. Q2, R2 and accuracy were calculated via double cross validation procedure with tenfold-CV, non-independent set was used.

### Mitochondrial dysregulation as a feature of severe COVID-19

To identify the disturbed pathways between the study groups, enrichment analysis (Metaboanalyst v4.0) employing the metabolites with the highest VIP values from PLS-DA analysis was applied; pathways with *p* < 0.05 are shown in Table 4. Pathways associated with the metabolism of BCAA, glutamate and phenylalanine were differentially expressed in patients with mild and severe COVID-19 as well as in controls. Additionally, we observed a possible involvement of the Warburg effect, as this process is differentially regulated in COVID-19 patients bearing different disease severity in comparison with controls. Further, differential pathways analysis among COVID-19 patients identified a potential mitochondrial dysregulation in those with severe disease. We also found that BCAA metabolism was differentially expressed in the studied groups, and glutamine and glutamate metabolism exhibited a high impact on the metabolic profile of COVID-19 patients (Fig. S2).

**Table 4:** Enriched pathway analysis identified in COVID19 patients.

Groups	Enriched metabolite set	Hits	*p* value	Holm P	FDR
C versus M	Glycine and serine metabolism	5	0.00103	0.101	0.0547
	Valine, leucine and isoleucine degradation	5	0.00112	0.108	0.0547
	Propanoate metabolism	4	0.00241	0.231	0.0786
	Phenylalanine and tyrosine metabolism	3	0.00669	0.636	0.118
	Urea cycle	3	0.0074	0.695	0.118
	Warburg effect	4	0.00792	0.737	0.118
	Malate-aspartate shuttle	2	0.00843	0.775	0.118
C versus S	Glutamate metabolism	3	0.0312	1.0	0.874
	Glutathione metabolism	2	0.0358	1.0	0.874
	Warburg effect	3	0.0483	1.0	0.874
M versus S	Valine, leucine and isoleucine degradation	4	0.00686	0.672	0.392
	Citric acid cycle	3	0.00799	0.775	0.392
	Glutathione metabolism	2	0.0314	1.0	0.655
	Transfer of acetyl groups into mitochondria	2	0.0343	1.0	0.655
	Warburg effect	3	0.0402	1.0	0.655
	Glycine and serine metabolism	3	0.042	1.0	0.655
	Cysteine metabolism	2	0.0468	1.0	0.655
C versus M versus S	Glutamate metabolism	3	0.0312	1.0	0.793
	Glutathione metabolism	2	0.0358	1.0	0.793
	Warburg effect	3	0.0483	1.0	0.793

Results of the enrichment pathways according to Metaboanalyst (v4.0), employing the VIP values from PLS-DA analysis as the input metabolites.

### Correlation of a distinct metabolic profile with ventilatory parameters and diverse clinical features

**Figure 3:**
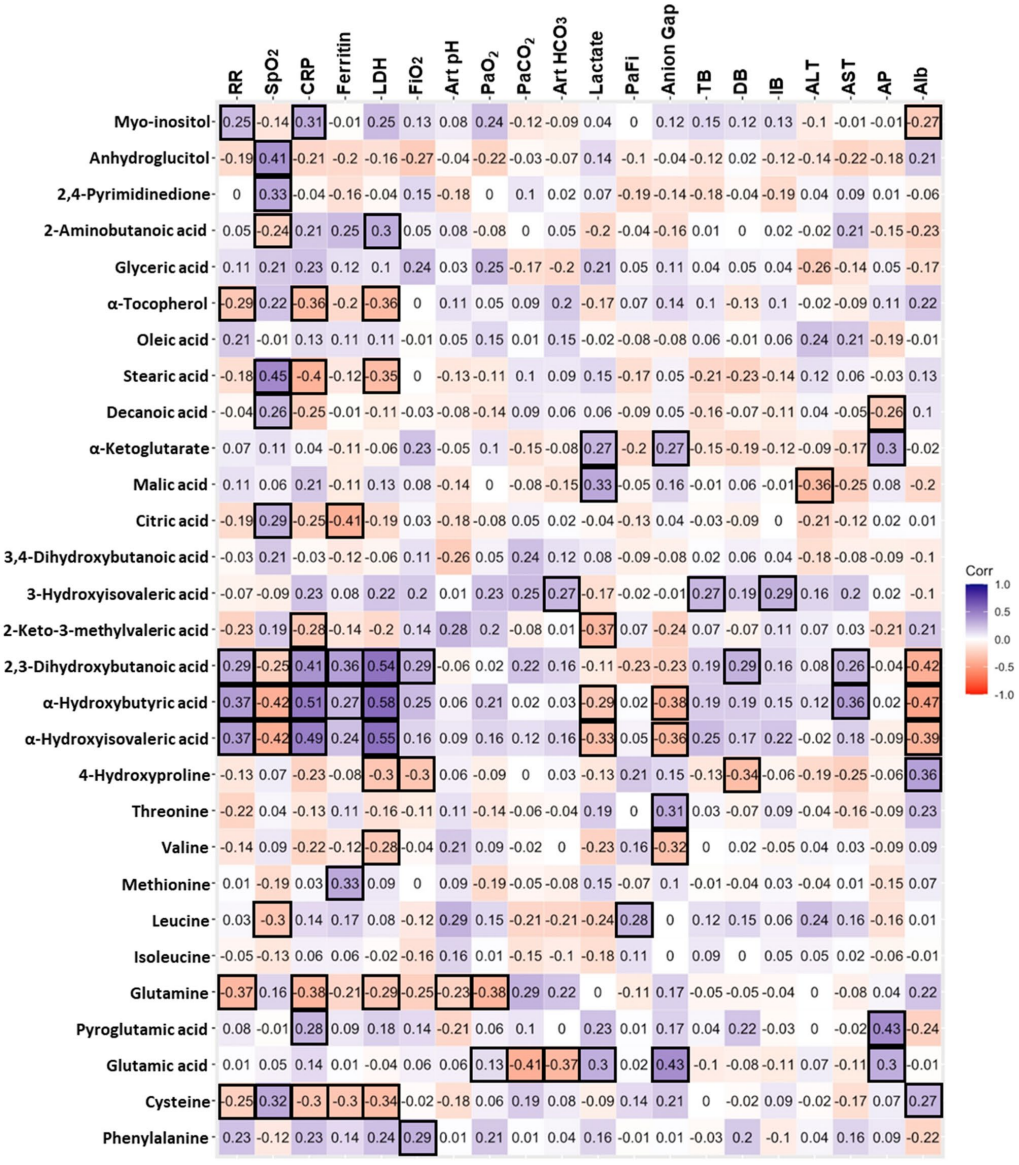
Spearman’s correlation analysis. Correlation analysis of the modified metabolites in severe COVID-19 patients and the clinical parameters considered. Blue numbers indicate a positive association. Red numbers denote a negative association. Squared values denote associations with values *p* < 0.05. RR = Respiratory rate, SpO_2_ = Oxygen saturation, LDH = Lactate dehydrogenase, FiO_2_ = Fraction of inspired oxygen, Art pH = Arterial pH, PaO_2_ = Partial pressure of oxygen, PaCO_2_ = Partial pressure of CO_2_, Art HCO_3_ = Arterial bicarbonate, PaFi = PaO_2_/ FiO_2_, TB = Total bilirubin, DB = Direct bilirubin, IB = Indirect bilirubin, ALT = Alanine aminotransferase, AST = Aspartate aminotransferase, AP = Alkaline phosphatase, Alb = Albumin. Exact *p* values are referred in Supplemental Table 2.

We performed a Spearman’s correlation analysis between VIP metabolites with routine clinical tests for oxygen homeostasis and clinical markers recently described for lung impairment and poor prognosis in SARS-CoV-2 infection. Figure 3 and Table S2 show significant correlations between metabolites and clinical variables. A generalized linear mixed models to examine the effect of patient condition (H, M or S) over VIP metabolites levels, including the presence of obesity and hypertension as random factors (Supplemental Table S3), revealed that only oleic acid did not show significant differences derived from the patients’ condition (HvsMvsS). For generalized linear models with age as a covariate, only 3-Hydroxyisovaleric acid, anhydroglucitol and threonic acid are influenced due to the patient age. Nonetheless, this variable does not replace the effect of COVID-19 (Supplemental Table 4). Furthermore, after adjustment, we also found that BMI influences the levels of 3-Hydroxyisovaleric and citric acid. However, these changes are explained based on the composite effect of BMI and COVID-19 status as well.

## Discussion

We identified altered levels of amino-acids, Krebs cycle intermediaries, and α-HA in serum samples from mild and severe COVID-19 patients. The analysis of these metabolic profiles revealed a relationship between an altered metabolism of BCAA, glutamate, glutamine, and Warburg effect with the severity of the disease. In particular, hypoxemia in COVID-19 patients could impair redox, energetic, and immune responses^1^. In this context, the term *metabolic flexibility* refers to the transcriptomic, proteomic and metabolic changes that need to be adapted to particular pathological conditions^10^. Globally, hypoxia favors anaerobic glycolysis (over beta oxidation, pentose phosphate pathway and cellular respiration) as a source of ATP to fulfill the energetic requirements of several critical processes such as RNA expression, and protein and lipid synthesis^11^.We detected modified levels of three intermediaries of the Krebs cycle. Citrate serum levels fell in severe COVID-19 patients and positively correlated with lower oxygen saturation levels. On the other hand, α-ketoglutarate and malate transiently increased in mild COVID-19 patients, showing a positive correlation with lactate and anion gap markers, both indicators of metabolic acidosis. Although it is unclear whether the degree of these changes were caused by the hypoxic conditions, the analysis of the differential metabolic signatures exhibited by mild and severe COVID-19 patients suggests that citric cycle and Warburg effect play a significant role for discriminating both groups. In normoxia, glucose conversion to acetyl Co-A and glutamine to α-ketoglutarate are the major sources of mitochondrial carbon intermediaries for the Krebs cycle^12^. However, in hypoxia, the flux of acetyl-coA is inhibited and glutamine-derived α-ketoglutarate can be reductively carboxylated to isocitrate by isocitrate dehydrogenase as a way to replenish mitochondrial NAD levels and increase citrate pools in the Krebs cycle^12,13^. This process has been extensively studied in cancer-derived hypoxia^14^. Thus, citrate may diffuse to cytosol where it could be further processed by the enzyme ATP citrate lyase to Acetyl-Co-A and oxaloacetate^15^. The cytosolic activity of malate dehydrogenase also might support an enhanced glycolysis through the conversion of oxaloacetate to malate, regenerating NAD^6^. In addition to the potential inhibition of the Krebs cycle, ATP citrate lyase activity could also explain the altered levels of citrate in mild COVID-19 patients.

We observed reduced levels of glutamine in severe and mild COVID-19 patients and this change negatively correlated with lactate dehydrogenase (LDH), C reactive protein (CRP), and PO2 levels, and positively with PCO2; these markers have been associated with lung damage and altered oxygen homeostasis in COVID-19 patients^16,17^. In vitro experiments also have demonstrated that hypoxia increases glutamine transport into the cells through HIF2-alpha upregulation of SLC1A5 genes^18^. These interconnected processes might potentially link glutamine hypoxic catabolism with NAD recycling and malate synthesis. According to our results, several reports detected reduced levels of this amino acid in COVID-19 cohorts, notwhitstanding, little is understood about this decrement and its potential therapeutic implications^7,8,19^. Further studies are needed to more precisely assess whether glutamine is linked to reductive carboxylation in hypoxic patients.

On the other hand, three different α-HA of amino acid origin (α-Hydroxyisovaleric acid, α-Hydroxybutyric, and 2,3-Dihydroxybutanoic acid) were significantly increased in samples from mild and severe COVID-19 patients and positively correlated with CRP and LDH and negatively with SO2 and serum albumin. Reduced levels of this protein have been associated with increased mortality in severe hypoxic hepatitis and COVID-19^20–22^. Similar to glutathione, albumin has an antioxidant activity over ROS control in ischemic and hypoxic liver^23^. The association between increased α-HA and lower albumin levels may be related to the decline in protein synthesis and modification of amino acid metabolism due to hypoxia^24,25^. In this regard, α-Hydroxyisovaleric acid is a product of valine catabolism and is used as a marker of maple syrup urine disease (MSUD), a clinical condition derived from inactivating mutations in branched-chain α-keto acid dehydrogenase complex (BCKDH), an enzyme essential for BCAA catabolism^26^. BCAA that are not required for protein synthesis are deaminated to α-KA (producing glutamate from α-ketoglutarate) by branched chain aminotransferases (BCAT) and funneled to mitochondrial BCKDH, where they are further metabolized in the Krebs cycle^27^. However, in hypoxic conditions, the halt of respiratory chain promotes an increase in NADH/NAD ratio, inactivating BCKDH complex and boosting α-KA levels^28^. Subsequently, cellular peroxidases oxidizes the resultant α-KA to α-HA in a NADH dependent manner^29,30^. As in MSUD, α-KA and α-HA are increased in patients with respiratory diseases that disturb oxygen homeostasis, and this increment is probably related to BCKDH inhibition and α-Keto oxidase activity^28,31,32^.

We identified as well modified serum valine, leucine, isoleucine, 2-keto-3-methylvaleric acid, and 3-Hydroxyisovaleric acid levels, which indeed are included in the BCAA metabolic pathway. These changes could suggest a modified BCAA metabolism in COVID-19 patients. Noteworthy, we also identified a trend towards restoration of the levels of these amino acids in patients with severe COVID-19 when compared to those with mild disease. In fact, 4-Hydroxyproline, a marker of amino acid mobilization from skeletal muscle to liver, displayed a trend towards higher levels in severe COVID-19 patients, suggesting that in severe disease, there is an increased requirement of tissue BCAA in order to replenish NAD.

Threonine levels decreased in severe and mild COVID-19 patients; this decrement could be potentially related to an enhanced catabolism as we also identified increased levels of α-Hydroxybutyric and 2,3-Dihydroxybutanoic acids, both oxidized metabolites of threonine conversion to α-HA. α-Hydroxybutyric acid is synthetized by the activity of lactate dehydrogenase over α-Ketobutyrate, a product of methionine/threonine catabolism and cysteine anabolism^33^. The increase of this metabolite correlated positively with higher levels of serum LDH. In this context, given that α-Ketobutyrate is a substrate of BCKDH, the increase of its corresponding α-HA could be related with the potential inactivity of the mitochondrial enzymatic machinery^34,35^. On the other hand, 2,3-Dihydroxybutanoic acid is a product of threonine catabolism probably generated by the activity of cytosolic transaminases and oxidases, a conversion that may potentially restore NAD during hypoxia^36^. All the enzymes involved in α-KA oxidation rely on NADH (or NADPH) as a co-substrate and their activities are important to maintain the energetic and redox states in health and disease. However, the mechanisms involved in BCAA and threonine intermediaries in hypoxia are still incompletely understood^37^.

A mechanism that links HIF1-alpha with the increase in BCAA transport and aminotransferase activity in hypoxic glioma cells was recently described^38^. In this cell context, the in vitro accumulation of BCAA in cerebral cortex of normoxic rats promotes glucose internalization and, interestingly, induces lower levels of CO2 release. These data suggest the presence of aerobic or anaerobic glycolysis regulated by the BCAA levels despite the presence of normoxia^39^. Interestingly, BCAT exhibits a CXXC motif that presumably acts as redox sensor and predisposes its aminotransferase activity by the cellular redox state^40^.

**Figure 4:**
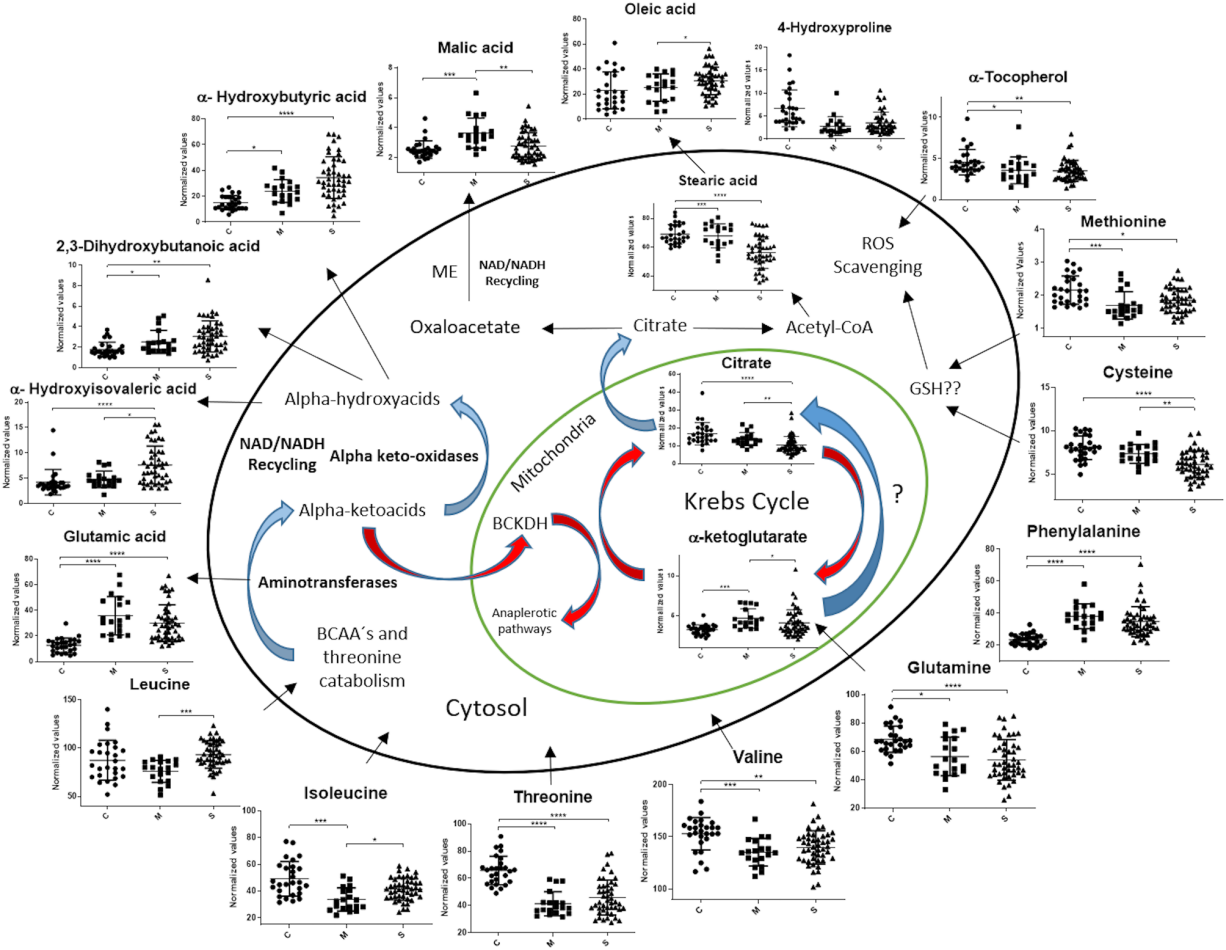
Proposed model for the serum metabolic changes observed in COVID-19 patients. Red arrows represent metabolic flux inhibited during hypoxia. Blue arrows represent increased flux in hypoxia. ME Malic enzyme. The graphs were constructed with the normalized values from control subjects (C), mild (M), and severe (S) patients. Asterisks indicate statistical significance according to the Dunn test. p* < 0.05 ** < 0.01, *** < 0.001, **** < 0.0001.

Although the effects of BCAA metabolism in hypoxia are still poorly understood, an evolutive advantage cannot be ruled out. In this regard, cells from the fungus *Aspergillus nidulans* increase the synthesis of BCAA as a mechanism to regenerate NAD and NADP during hypoxia^41^. Interestingly, the in vitro addition of BCAA, such as valine and leucine, rises the survival rate of mice infected with *Klebsiella Pneumoniae*^42^. Our present data suggest that hypoxia promotes the metabolic funneling of BCAA and threonine to α-KA synthesis and then to α-HA as a compensatory mechanism to replenish NAD levels in COVID-19 patients (Fig. 4).

In support to this hypothesis, we observed decreased serum levels of BCAA and threonine in COVID 19 patients, an effect that has been previously identified in several studies of chronic obstructive pulmonary disease^37^. It has been shown that BCAA mobilization from skeletal muscle to liver is essential for human adaptability to lower oxygen levels^43^, which is in agreement with our findings. Recent data highlight the relevance of BCAA administration as an strategy to attenuate protein muscle loss in COVID-19 critical patients^44^.

An scenario with high amino transferase activity requires a constant flux of α-Ketoglutarate, a nitrogen acceptor, which could be achieved through an enhanced glutamine catabolism^12^. In this regard, α-KA synthesis mediated by aminotransferases generates glutamate as a byproduct. Accordingly, we observed an increase in glutamic acid levels, which correlated positively with anion gap values in severe COVID-19. Glutamate is an important neurotransmitter that, if unbalanced, promotes several neurological abnormalities^45,46^. In fact, patients with hypoxia may exhibit impaired activity of glutamate and several other neurotransmitters involved in homeostasis of the central nervous system, which may explain some of the neurological features found in COVID-19 patients^47^.

On the other hand, the appearance of new onset or worsening of pre-existing diabetes has been identified in COVID-19 patients^48^. α-Hydroxybutyric acid is an early marker of insulin resistance in the non-diabetic population^49,50^. Additionally, high levels of 2,3- Dihydroxybutanoic acid and α-Hydroxybutyric acid were detected in type I diabetes patients^36^. Gall et al^54^ proposes that individuals with an augmented fatty acid metabolism display NADH/NAD imbalance resembling hypoxic conditions that unleash a change in BCAA metabolic fate, predisposing to diabetes development. Although there are still missing paths connecting BCAA metabolism with diabetes, a plausible explanation for the presence of insulin resistance in COVID-19 patients may be partly connected to BCKDH state^51^.

## Conclusion

Three different α-hydroxyl-acids are increased in severe COVID-19. The underlying biochemical traits may involve a modified amino acid metabolism derived from lung damage and hypoxic conditions. It is tempting to propose that the enzymatic conversion of α-keto-acids to α-hydroxyl-acids helps to maintain NAD recycling in patients with altered oxygen levels, highlighting the potential relevance of amino acid supplementation during SARS-CoV-2 virus infection.

## Methods

### Subjects

Sixty-five COVID-19 patients, confirmed by a positive RT-PCR test for SARS-CoV-2 on a nasopharyngeal swab, and 27 control donors with a negative PCR test for SARS-CoV-2 were recruited at a third level referral center in Mexico City (Instituto Nacional de Ciencias Médicas y Nutrición Salvador Zubirán) from March to June, 2020.

Upon patient assessment, general laboratory tests were performed (complete blood count, glucose, blood urea nitrogen (BUN), creatinine (Cr), liver function tests, C-reactive protein (CRP), lactate dehydrogenase (LDH), creatinine kinase (CPK), fibrinogen, D-dimer, coagulation tests and ferritin). The severity of the disease was classified as follows: Mild/Moderate illness: Fever, signs of airway disease, with or without a tomographic image indicating pneumonia. Severe illness, any of the following: respiratory failure, respiratory rate > 30 bpm, O_2_ saturation < 92% at rest, PaO_2_/FiO_2_ < 300 mmHg^52^ . Once obtained, the serum was processed immediately and frozen to − 80 °C until GC/MS analysis, which was performed following a randomized scheme in the same way as COVID-19 samples. All samples from subjects included were drawn upon admission to the triage at the emergency department for the patients and at the epidemiology unit where the hospital staff attended for randomized nasopharyngeal PCR testing, during the morning shift and in a fasting state (minimum 8 h). The study was approved by the institutional ethics and research committees of Instituto Nacional de Ciencias Médicas y Nutrición Salvador Zubirán (Ref. 3341). In those subjects that met the inclusion criteria, serum samples were collected from an antecubital vein and stored at − 80 °C until the day of metabolomics processing. Written informed consent to participate in the study was obtained from all participants. All analytical methods were carried out in accordance with relevant guidelines and regulations.

### Gas chromatography/mass spectrometry (GC/MS) analysis

Forty-five microliters of serum sample and 10 µL of internal standard (IS—tetradecanoic acid, methyl tricosanoate, 5α-cholestane—0.36 mg/mL) were mixed in 150 µL of 1:3 chloroform–methanol and thoroughly mixed for 2 min. Samples were incubated at − 20 °C for 20 min and then centrifuged at 14,000 rpm for 15 min. One-hundred and fifty microliters of recovered supernatants were dried under nitrogen flow. Each precipitate was redissolved in 20 uL of methoxyamine and incubated for 90 min at 37 °C in a shaking incubator. Thereafter, 40 µL of MBSTFA + 1% TMCS was added to each sample and incubated for 30 min at 37 °C. One microliter was applied to a GC/MS system (Agilent 5977A/7890B, Santa Clara, CA, USA) with an automatic autosampler (G4513A, Agilent) and run under the following conditions: splitless column flow 1 ml/min, inlet temperature 200 °C, EI source temperature 200 °C, and interface temperature 250 °C. A column HP5ms (30 m × 250 µm × 0.25 µm, Agilent) with helium 99.9999% as a mobile phase was employed. The running method consisted of 1 min hold at 60 °C with an increased ramp of 10 °C/min to 325 °C, with a final held time of 10 min.

Briefly, to assess the system suitability along all the analysis we employed three different internal standards (IS). To identify any issues in the derivatization process, we employed tridecanoic acid as a metabolite prone to derivatization. To check shifts over the retention times we included additionally 5-alpha cholestane and tricosanoic acid methyl ester. The RSD values for the IS over all the experimental process (encompassing 104 samples in 12 batches = 12 days of analysis) were 12.89, 10.78 and 11.57% respectively (before the normalization process). For the intraday variation, we injected a QC1 (consisting of equal volumes of all the samples of the batch) every 5 samples along the entire batch. We observed an average %RSD value of 17.7% (the average of all metabolite RSD values (triplicate) in each batch [12 batches]). For the inter-day variation, we generated a QC2 consisting of ten aleatory selected samples. We observed an inter-day RSD value of 17.5% (average of RSD values for each metabolite in 12 batches). The %RSD values for each metabolite are shown in supplementary Table 1. These results are in accordance with Fiehn Laboratory recommendations for metabolomic analysis (< 30% RSD)^53^.

### Deconvolution and identification

GC/MS data was transformed to .mzdata using the Agilent Chemstation software (Agilent). Feature detection, spectral deconvolution and peak alignment were performed using the Mzmine2 software^54^. The parameters used were: RT range, 5.5–27.5 min; m/z range, 50–500; m/z tolerance, 0.5; noise level, 1 × 10^3^; and peak duration range, 0.01–0.2 min. Further metabolite selection was performed according to the rule of 80%. Identifications were determined using the National Institute of Standards and Technology (NIST) 2.0 spectral library. Results higher than 70% (R > 700), were accepted as correct, while values below this limit were marked as unknown and omitted. Only identified metabolites with %RSD lower than 30% were selected to further uni- or multivariate statistical analysis.

### Statistical analysis

Peak heights raw data was normalized by sum, log-transformed and autoscaled (mean-centered and divided by the standard deviation) for statistical multivariate analysis. PLS-DA and hierarchical cluster analysis were performed by the Ward’s method using *Metaboanalyst 4.0*^55^. For univariate analysis, data were normalized by sum and then the Kruskal–Wallis test was applied to examine the three levels of patient condition (control, moderate, and severe), followed by the Dunn’s post hoc test and unpaired Mann–Whitney test to examine differences between mild and severe conditions. For these analyses, the GraphPad Prism version 6 software (GraphPad Software Inc., San Diego, CA) was employed. *p* values < 0.05 were considered significant. Spearman’s correlation analysis, generalized linear models and generalized linear mixed models were performed in R version 4.0.2 (R Foundation for Statistical Computing).

### Metabolic pathway analysis: Enrichment analysis

The VIP values from PLS-DA analysis were selected to implement the enrichment pathway analysis in Metaboanalyst (v.4.0). In the case of α-Hydroxyisovaleric acid, the corresponding α-KA was used as the input metabolite. KEGG database does not have the correspondent α-HA derived of BCAA’s catabolism in its library. Only the enrichment analysis with *p* values < 0.05 are presented.

## Supplementary Information

Supplementary Information.
