# Supplementary material for: Metabolomics analysis reveals a modified amino acid metabolism that correlates with altered oxygen homeostasis in COVID-19 patients
Source: Sci Rep. 2021 Mar 18;11:6350. doi: 10.1038/s41598-021-85788-0 (PMC7973513; doi:10.1038/s41598-021-85788-0)
Supplement: Supplementary file 1 — Supplementary Information. [file 41598_2021_85788_MOESM1_ESM.docx]

Metabolomics analysis reveals a modified amino acid metabolism that correlates with altered oxygen homeostasis in COVID-19 patients

José C. Páez-Franco, José J. Torres-Ruíz, Víctor A. Sosa-Hernandez, Rodrigo Cervantes-Díaz, Sandra Romero-Ramírez, Alfredo Pérez-Fragoso, David E. Meza-Sánchez, Juan Manuel Germán-Acacio, José L. Maravillas-Montero, Nancy R. Mejía-Domínguez, Alfredo Ponce-de-León, Alfredo Ulloa-Aguirre, Diana Gómez-Martín and Luis Llorente

**Supplementary material**

**Supplemental Figure S1. Principal component analysis of all patients included in the analysis**.

**
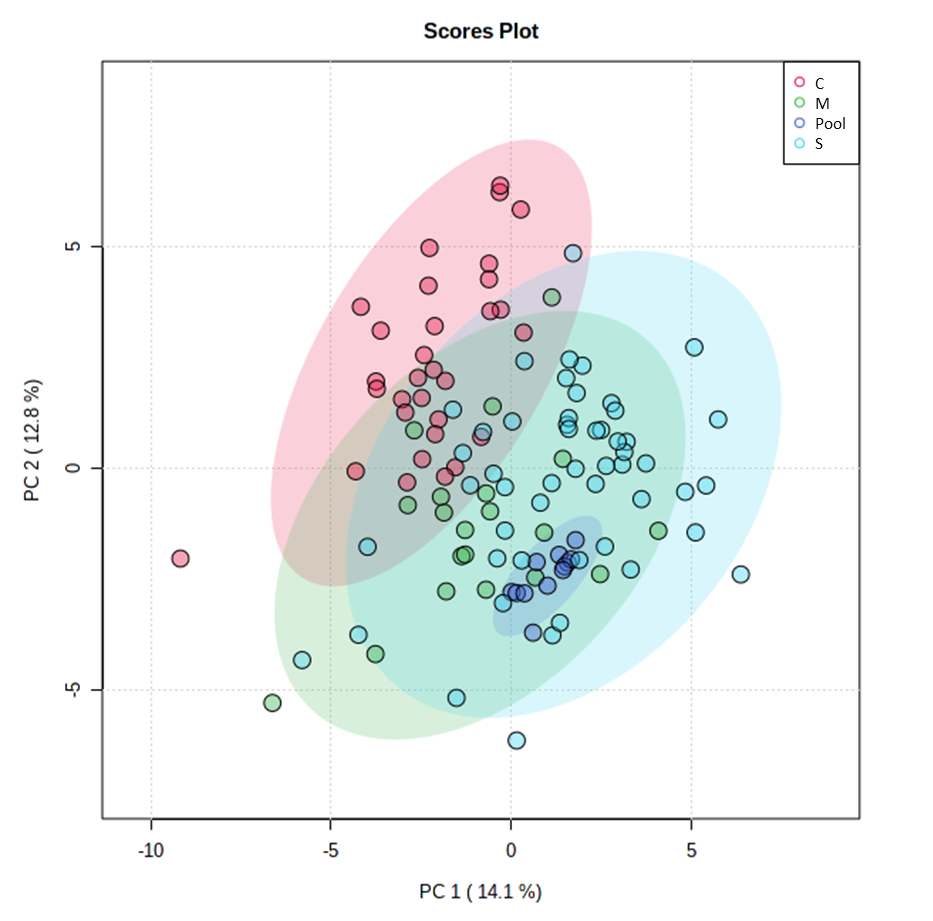
Figure S1. Principal component analysis of all patients included in the analysis**. Pooled samples (blue intense) were used as a QC and shows a compact well-defined cluster, ensuring a good reproducibility of our GC/MS analysis.

**Supplemental Figure S2. Pathway mapping based on VIP metabolites from PLS-DA analysis**

**
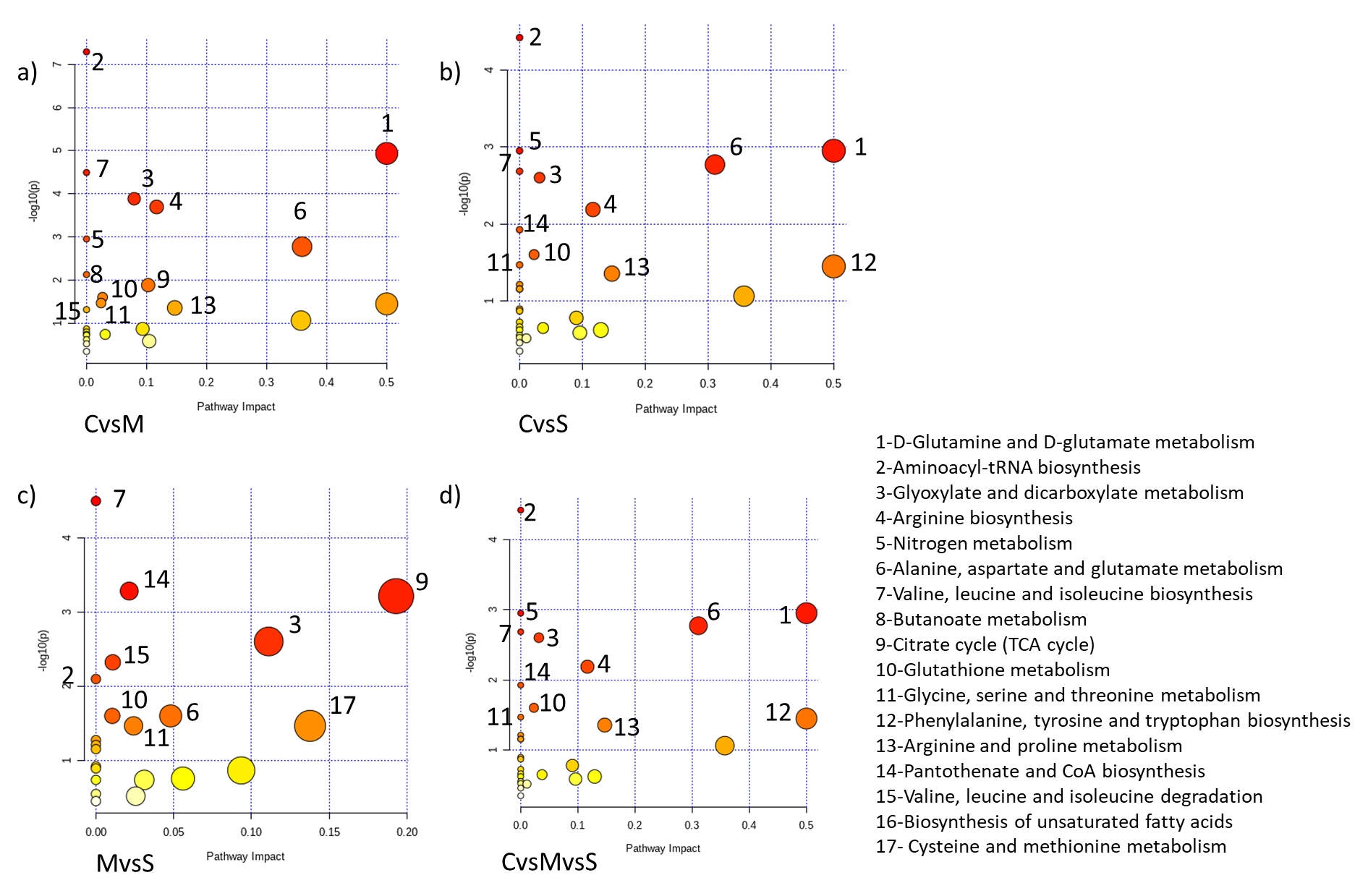
Figure S2. Pathway mapping based on VIP metabolites from PLS-DA** for A)CvsM, B) CvsS, C) MvsS and D) CvsMvsS combinations. Only the pathways with p<0.05 are marked.

**Supplemental Figure S3 - Scatter plots showing correlation between metabolite levels and clinical and laboratory parameters. P Spearman values are described in Supplementary Table 2.**


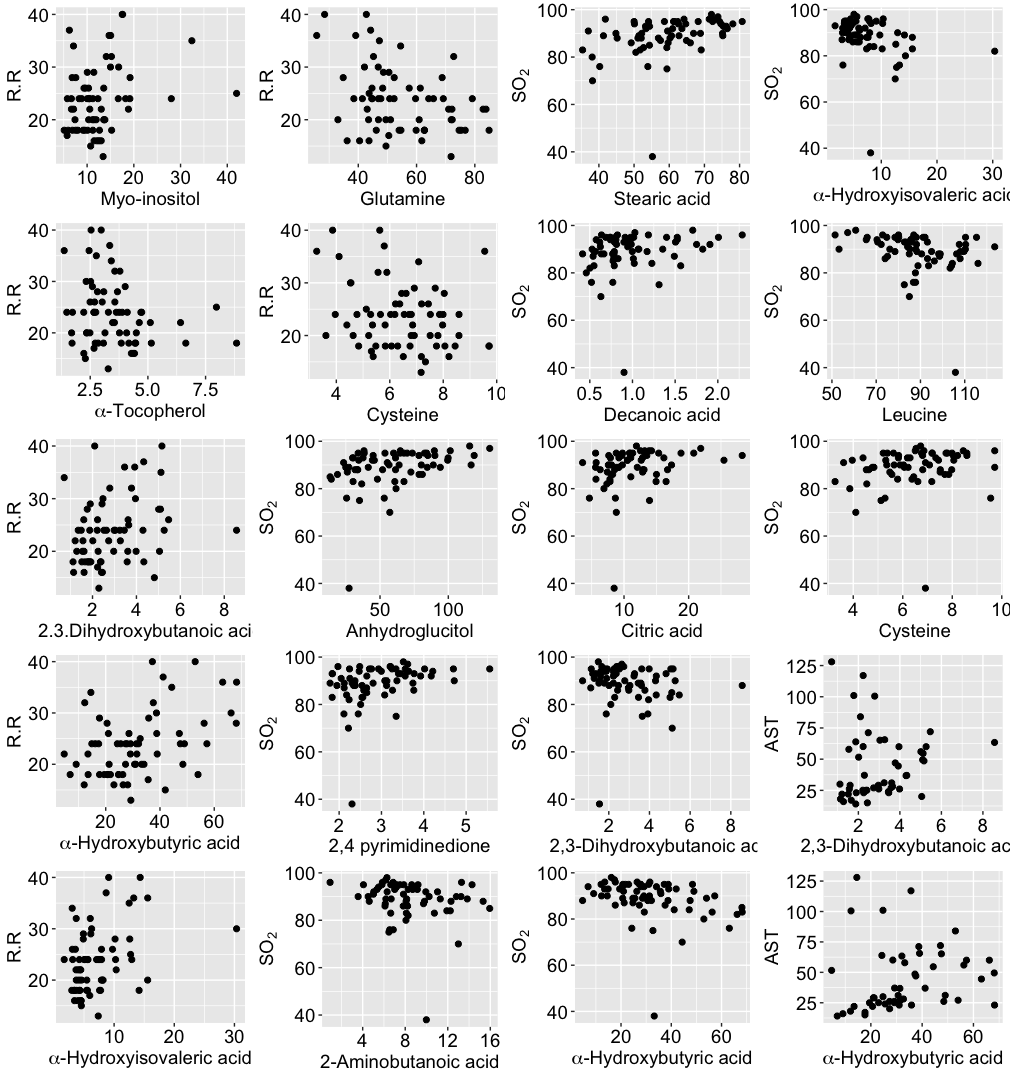


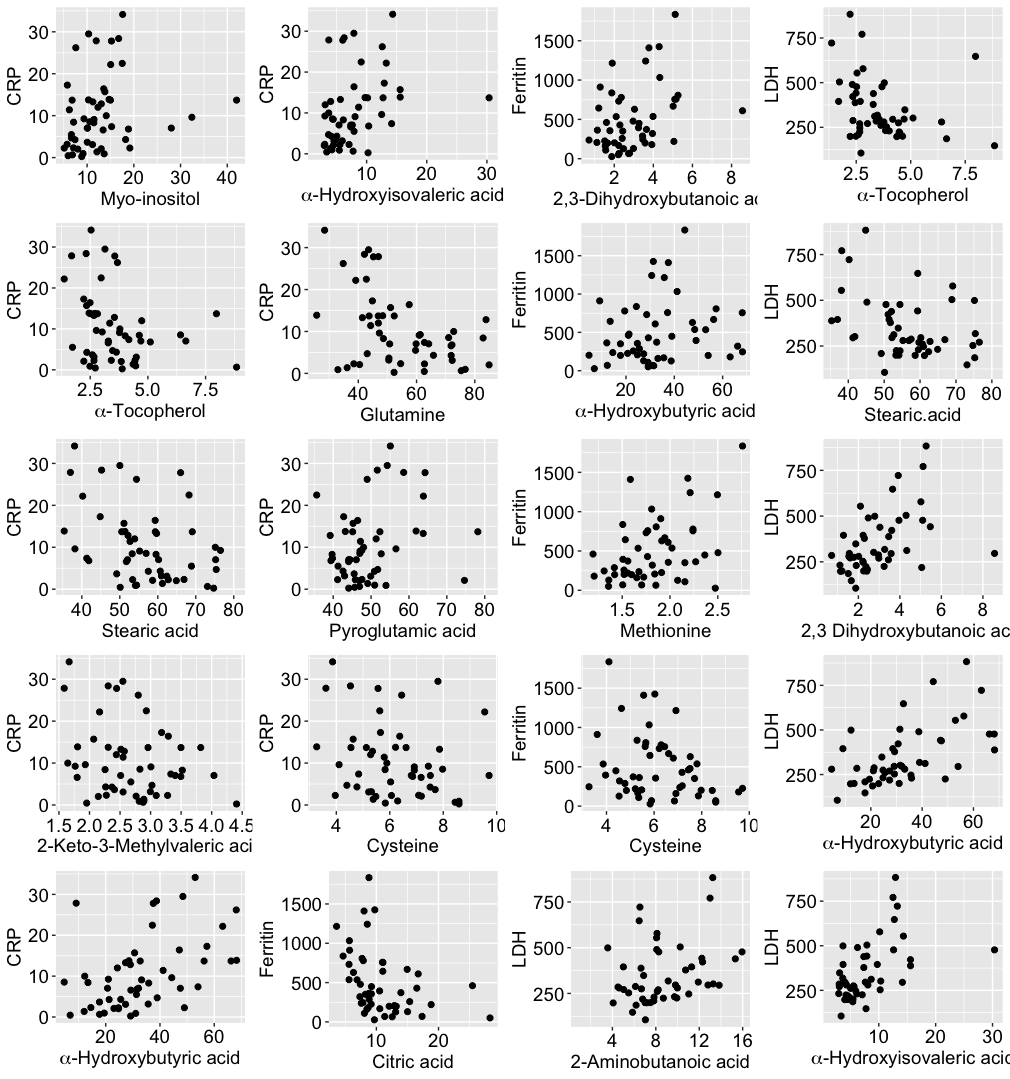


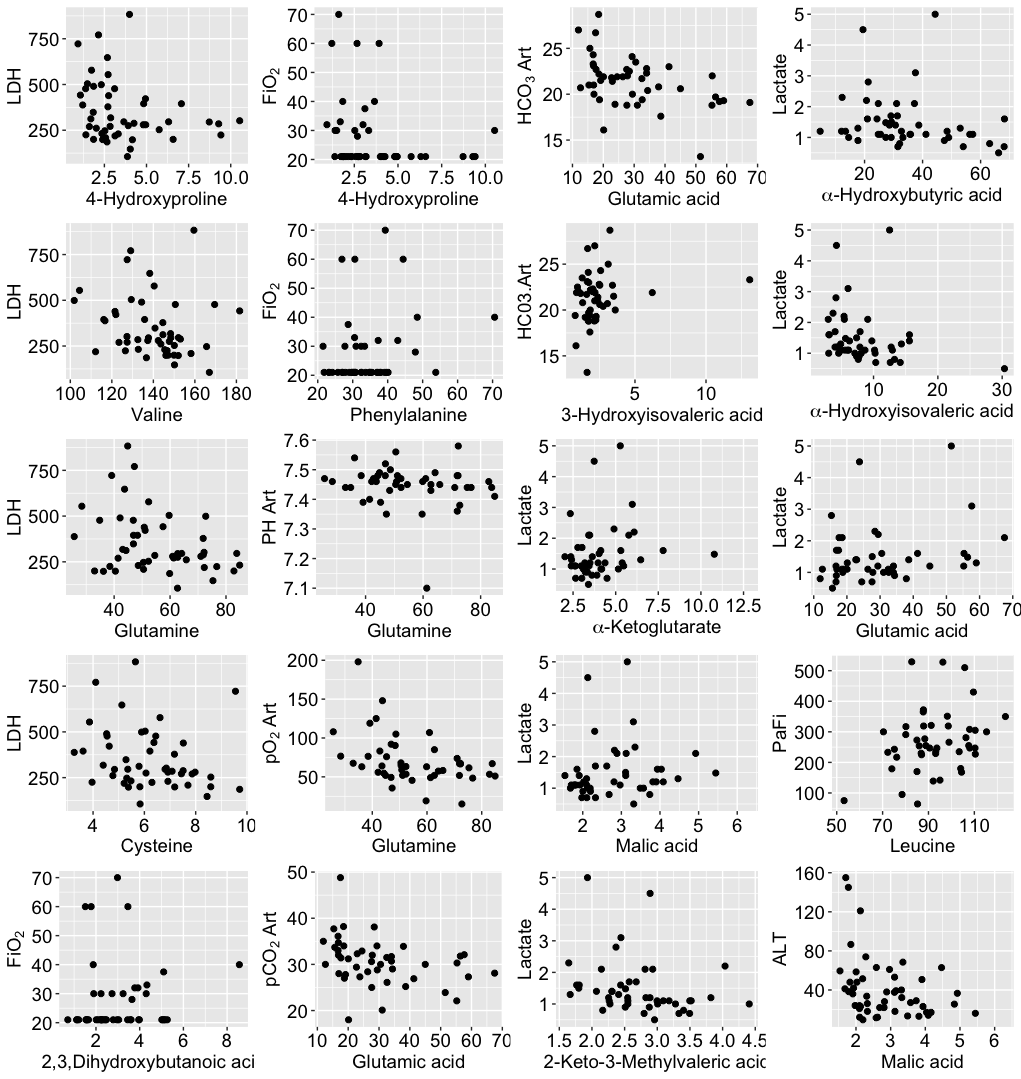


**Figure S2 - Scatter plots showing correlation between metabolite levels and clinical and laboratory parameters. P Spearman values are described in Supplementary Table 2.**

**Supplemental table 1. List of metabolites identified in our GC/MS analysis.**

| **Metabolite** | **HMDB** | **m/z** | **Retention time** | **Match %** | **%RSD** | **Kruskal-Wallis CvsMvsS**  **p.adjusted** | **Dunn**  **Post-Hoc**  **Test** | **Fold Change/log2(FC)**  **MvsC**  **SvsC**  **SvsM** | **Mann Whitney**  **p.adjusted**  **MvsC**  **SvsC**  **SvsM** |
| --- | --- | --- | --- | --- | --- | --- | --- | --- | --- |
| **2,3-Dihydroxybutanoic acid** | **HMDB02453** | **73.0** | **10.61** | **67.3** | **14.63** | **M - C; S – C**  **0.00024421** | **C-M * Up**  **C-S **** Up** | **1.4221/0.50807**  **1.7324/0.7928**  **1.2182/0.28473** | **0.0072403**  **5.73X10^-5^**  **0.29268** |
| **2,4-Pyrimidinedione** | **HMDB02453** | **73.0** | **12.44** | **70.2** | **15.26** | **M - C; C - S; M – S**  **0.0003393** | **C-S **** Down** | **1.1558/0.20887**  **0.88982/-0.16842**  **0.76988/-0.37729** | **0.030505**  **0.017826**  **0.00092486** |
| **2-Aminobutanoic acid** | **HMDB0000076** | **73.0** | **8.09** | **82** | **19.23** | **S - C; S – M**  **0.0094238** | **C-S * Down** | **0.98373/-0.02367**  **1.3022/0.38093**  **1.3237/0.4046** | **0.79567**  **0.010737**  **0.053197** |
| **2-Keto-3-methyl valeric acid** | **HMDB0000491** | **73.0** | **8.48** | **78.5** | **22.62** | **C - M; C – S**  **0.0027263** | **C-M ** Down**  **C-S ** Down** | **0.81825/-0.28938**  **0.83846/-0.25419**  **1.0247/0.035195** | **0.0020752**  **0.0052581**  **0.83453** |
| **2-Ketoisocaproic acid** | **HMDB0000695** | **75.0** | **8.66** | **81.9** | **24.18** | **0.16201** |  | **0.83193/-0.26546**  **0.89062/-0.16712**  **1.0705/0.098336** | **0.33921**  **0.065174**  **1** |
| **2-Piperidone** | **HMDB0011749** | **73.0** | **11.89** | **84.1** | **15.83** | **S - C; S – M**  **0.1002** |  | **0.98423/-0.022927**  **1.1483/0.19948**  **1.1667/0.2224** | **0.81003**  **0.11733**  **0.11495** |
| **3,4-Dihydroxybutanoic acid** | **HMDB0000337** | **73.0** | **11.60** | **74.4** | **11.40** | **0.013974** | **C-M ** Up** | **1.2716/0.34667**  **1.2257/0.29365**  **0.96392/-0.053015** | **0.0033236**  **0.31639**  **0.087626** |
| **3-Hydroxybutiric acid** | **HMDB0000011** | **75.0** | **7.92** | **70.1** | **18.25** | **S - C; S – M**  **0.012532** | **C-S *Up** | **1.0702/0.097832**  **1.8611/0.89619**  **1.7391/0.79835** | **0.51469**  **0.011845**  **0.087626** |
| **3-Hydroxyisovaleric acid** | **HMDB0000754** | **102.7** | **8.58** | **89.7** | **19.65** | **M - C; S – C**  **0.0018499** | **C-M * Up**  **C-S *** Up** | **1.5081/0.59274**  **1.6859/0.75355**  **1.1179/0.16081** | **0.02247**  **0.00063424**  **0.83453** |
| **4-Hydroxyproline** | **HMDB0000725** | **230.1** | **12.79** | **92.3** | **13.97** | **C - M; C – S**  **5.47X10^-5^** | **C-M**** Down**  **C-S***Down** | **0.44838/-1.1572**  **0.57316/-0.80298**  **1.2783/0.35423** | **3.04X10^-5^**  **0.00027734**  **0.24967** |
| **Anhydroglucitol** | **HMDB0002712** | **73.0** | **16.53** | **73.8** | **13.80** | **M - C; M – S**  **0.00095594** | **M-S*** Down** | **1.3004/0.37897**  **0.81825/-0.28939**  **0.62922/-0.66836** | **0.073608**  **0.065174**  **0.001212** |
| **Asparagine** | **HMDB0000168** | **73.0** | **14.49** | **93.6** | **28.44** | **0.24462** |  | **0.96178/-0.056216**  **0.9028/-0.14753**  **0.93867/-0.091309** | **0.53521**  **0.1255**  **0.58714** |
| **Cholesterol** | **HMDB0000067** | **75.0** | **26.96** | **83.9** | **12.19** | **0.080205** |  | **1.1405/0.18967**  **1.0234/0.033399**  **0.89734/-0.15627** | **0.045357**  **0.90514**  **0.089309** |
| **Citric acid** | **HMDB0000094** | **73.0** | **16.18** | **93** | **15.25** | **C - S; M – S**  **7.07X10^-6^** | **C-S **** Down**  **M-S** Down** | **0.81753/-0.29066**  **0.62276/-0.68324**  **0.76176/-0.39259** | **0.090738**  **3.21X10^-6^**  **0.0040909** |
| **Cysteine** | **HMDB0000574** | **73.0** | **13.14** | **81.7** | **22.21** | **C - S; M – S**  **1.01X10^-5^** | **C-S **** Down**  **M-S ** Down** | **0.91181/-0.13319**  **0.75897/-0.39788**  **0.83238/-0.26469** | **0.13803**  **3.21X10^-6^**  **0.0056437** |
| **Cystine** | **HMDB0000192** | **73.0** | **20.65** | **98.1** | **19.10** | **0.065335** |  | **1.055/0.07725**  **1.163/0.2178**  **1.1023/0.14055** | **0.49746**  **0.034841**  **0.27291** |
| **Decanoic acid** | **HMDB00511** | **73.0** | **11.79** | **90.4** | **16.25** | **C - S; M – S**  **0.00068398** | **C-S *** Down**  **M-S * Down** | **0.96249/-0.055153**  **0.72772/-0.45854**  **0.75608/-0.40338** | **0.53521**  **0.00023094**  **0.052317** |
| **Glutamic acid** | **HMDB0000148** | **246.1** | **13.88** | **93.5** | **15.73** | **M - C; S – C**  **4.96X10^-9^** | **C-M **** Up**  **C-S **** Up** | **2.811/1.491**  **2.3519/1.2338**  **0.83674/-0.25715** | **5.89X10^-9^**  **1.62X10^-9^**  **0.17658** |
| **Glutamine** | **HMDB0000641** | **156.0** | **15.61** | **97.7** | **14.56** | **C - M; C – S**  **0.00035244** | **C-M * Down**  **C-S **** Down** | **0.82234/-0.28219**  **0.78704/-0.34548**  **0.95708/-0.063296** | **0.013894**  **5.64X10^-5^**  **0.65016** |
| **Glyceric acid** | **HMDB0000139** | **73.0** | **10.29** | **89.5** | **16.50** | **M - C; M – S**  **0.00041509** | **C-M ** Up**  **M-S *** Down** | **1.3868/0.47179**  **0.98286/-0.024946**  **0.70871/-0.49674** | **0.00092556**  **0.79961**  **0.00066696** |
| **Glycine** | **HMDB0000123** | **102.0** | **7.37** | **94.3** | **25.22** | **0.99442** |  | **0.97838/-0.031534**  **0.98011/-0.028989**  **1.0018/0.0025449** | **1**  **0.90514**  **0.99141** |
| **Heptadecanoic acid** | **HMDB0002259** | **117.0** | **19.11** | **93** | **10.78** | **0.95182** |  | **0.92376/-0.11441**  **0.94953/-0.074717**  **1.0279/0.03969** | **0.81003**  **0.83556**  **0.96544** |
| **Hexadecanoic acid** | **HMDB0000220** | **117.0** | **18.21** | **98** | **13.92** | **0.71615** |  | **0.96875/-0.045806**  **1.0352/0.049928**  **1.0686/0.095735** | **0.95075**  **0.64455**  **0.57053** |
| **Hexanoic acid** | **HMDB0000535** | **75.0** | **6.73** | **90.9** | **18.16** | **0.18585** |  | **1.1249/0.16984**  **0.95424/-0.067577**  **0.84826/-0.23741** | **0.81003**  **0.13409**  **0.26824** |
| **Hypoxanthine** | **HMDB0000157** | **73.0** | **15.93** | **95.5** | **16.33** | **M - C; S – C**  **0.0094238** | **C-M * Up**  **C-S ** Up** | **1.3806/0.46528**  **1.4175/0.50331**  **1.0267/0.038027** | **0.021736**  **0.0077175**  **1** |
| **Isoleucine** | **HMDB0000172** | **158.1** | **9.77** | **77.5** | **20.51** | **C - M; C - S; S – M**  **0.00028952** | **C-M*** - Down**  **M-S * - Up** | **0.68594/-0.54385**  **0.84462/-0.24362**  **1.2313/0.30023** | **0.0001764**  **0.034063**  **0.0070619** |
| **Leucine** | **HMDB0000687** | **158.1** | **9.47** | **74.8** | **11.64** | **C - M; S – M**  **0.00069257** | **M-S*** Up** | **0.86945/-0.20183**  **1.0674/0.094071**  **1.2276/0.2959** | **0.14668**  **0.10416**  **0.00056823** |
| **Malic acid** | **HMDB0000156** | **73.0** | **12.32** | **89.9** | **10.95** | **M - C; M – S**  **0.00069257** | **C-M *** Up**  **M-S ** Down** | **1.4325/0.51851**  **1.0887/0.12256**  **0.75999/-0.39595** | **5.05X10^-5^**  **0.69902**  **0.0056437** |
| **Methionine** | **HMDB0000696** | **61.0** | **12.67** | **95.1** | **14.99** | **C - M; C – S**  **0.0010628** | **C-M *** Down**  **C-S * Down** | **0.78628/-0.34689**  **0.85599/-0.22434**  **1.0887/0.12255** | **0.00067223**  **0.012145**  **0.14326** |
| **Myo-inositol** | **HMDB0000211** | **305.1** | **18.95** | **82** | **14.66** | **M - C; S – C**  **0.00064644** | **C-M** Up**  **C-S** Up** | **1.3023/0.38109**  **1.4839/0.5694**  **1.1394/0.18832** | **0.0012658**  **0.00061801**  **0.83453** |
| **Oleic acid** | **HMDB0000207** | **117.0** | **19.78** | **72** | **12.85** | **S – C**  **0.045496** | **C-S * Up** | **1.0957/0.13185**  **1.3317/0.41325**  **1.2154/0.28139** | **0.53521**  **0.023221**  **0.24967** |
| **Palmitelaidic acid** | **HMDB0012328** | **55.0** | **17.93** | **70.2** | **16.48** | **0.15463** |  | **1.2341/0.30344**  **1.0473/0.066687**  **0.84866/-0.23675** | **0.084674**  **0.77237**  **0.17994** |
| **Phenylalanine** | **HMDB0000159** | **73.0** | **13.97** | **77.5** | **13.34** | **M - C; S – C**  **4.96X10^-9^** | **C-M **** Up**  **C-S **** Up** | **1.616/0.692**  **1.478/0.56361**  **0.91461/-0.12877** | **1.73X10^-8^**  **1.01X10^-9^**  **0.094519** |
| **Pyroglutamic acid** | **HMDB0000267** | **73.0** | **12.71** | **70.6** | **18.31** | **M - C; M – S**  **0.005863** | **C-M ** Up**  **M-S * Up** | **1.2155/0.28151**  **1.0592/0.083032**  **0.87147/-0.19848** | **0.0073589**  **0.25829**  **0.014877** |
| **Serine** | **HMDB0000187** | **204.1** | **10.68** | **93.6** | **17.72** | **0.25144** |  | **1.0827/0.11465**  **1.0833/0.11545**  **1.0006/0.00079758** | **0.1998}**  **0.16702**  **0.96544** |
| **Stearic acid** | **HMDB00827** | **73.0** | **19.99** | **89** | **14.68** | **C - S; M – S**  **7.07X10^-6^** | **C-S **** Down**  **M-S *** Down** | **0.98373/-0.023662**  **0.8153/-0.2946**  **0.82878/-0.27093** | **0.79567**  **3.21X10^-6^**  **0.0012704** |
| **Tetradecanoic acid** | **HMDB0000806** | **73.0** | **16.25** | **96.3** | **14.65** | **C - M; C – S**  **0.11971** |  | **0.73248/-0.44913**  **0.75478/-0.40586**  **1.0304/0.043269** | **0.14864**  **0.065174**  **0.99211** |
| **Threonic acid** | **HMDB0000943** | **73.0** | **13.08** | **77.6** | **13.54** | **M – S**  **0.002887** | **M-S ** Down** | **1.0659/0.092109**  **0.89581/-0.15873**  **0.84041/-0.25084** | **0.18901**  **0.065174**  **0.0031371** |
| **Threonine** | **HMDB0000167** | **73.0** | **11.04** | **97.8** | **17.27** | **C - M; C – S**  **8.59X10^-8^** | **C-M**** Down**  **C-S **** Down** | **0.62775/-0.67174**  **0.69792/-0.51887**  **1.1118/0.15286** | **6.68X10^-8^**  **5.32X10^-8^**  **0.30551** |
| **Uridine** | **HMDB0000296** | **73.0** | **21.93** | **83.3** | **21.40** | **0.13398** |  | **1.1623/0.21704**  **1.0958/0.13203**  **0.94278/-0.085013** | **0.084674**  **0.16056**  **0.54292** |
| **Valine** | **HMDB0000883** | **144.1** | **8.70** | **76.6** | **11.60** | **C - M; C – S**  **0.00041509** | **C-M *** Down**  **C-S** - Down** | **0.88451/-0.17705**  **0.91468/-0.12866**  **1.0341/0.048394** | **0.00067223**  **0.00091996**  **0.3267** |
| **α-Hydroxybutyric acid** | **HMDB0000008** | **147.0** | **7.49** | **79.3** | **19.30** | **M - C; S - C; S – M**  **8.59X10^-8^** | **C-M * Up**  **C-S**** Up** | **1.5999/0.67795**  **2.3095/1.2076**  **1.4436/0.52963** | **0.00092556**  **4.04X10^-8^**  **0.033291** |
| **α-Hydroxyisovaleric acid** | **HMDB0000407** | **73.0** | **8.00** | **73** | **17.07** | **S - C; S – M**  **1.71X10^-5^** | **C-S **** - Up**  **M-S* - Up** | **1.133/0.18019**  **1.9297/0.94836**  **1.7031/0.76817** | **0.024519**  **7.55X10^-6^**  **0.014705** |
| **α-Ketoglutarate** | **HMDB0000208** | **73.0** | **13.36** | **98.3** | **14.47** | **M - C; S - C; M – S**  **0.00060858** | **C-M *** Up**  **M-S * Down** | **1.5638/0.64508**  **1.2522/0.32445**  **0.80072/-0.32063** | **3.04X10^-5^**  **0.072374**  **0.041873** |
| **α-Tocopherol** | **HMDB0001893** | **73.0** | **26.79** | **73** | **28.68** | **C - M; C – S**  **0.0042218** | **C-M * Down**  **C-S ** Down** | **0.78333/-0.3523**  **0.7694/-0.37819**  **0.98222/-0.025889** | **0.02247**  **0.002085**  **1** |
| **Cholestane (IS)** |  | **81.0** | **24.92** | **71** | **10.78** |  |  |  |  |
| **Tricosanoic acid (IS)** |  | **74.0** | **23.09** | **87.7** | **11.57** |  |  |  |  |
| **Tridecanoic acid (IS)** |  | **73.0** | **15.23** | **94.7** | **12.89** |  |  |  |  |

**Supplemental table 1. List of metabolites identified in our GC/MS analysis.** %RSD was calculated with QC sample runs (generated from ten aleatory selected samples) injected every day of analysis. IS internal standard.

**Supplemental table 2. p-values of Spearman´s correlation analysis in image 3**


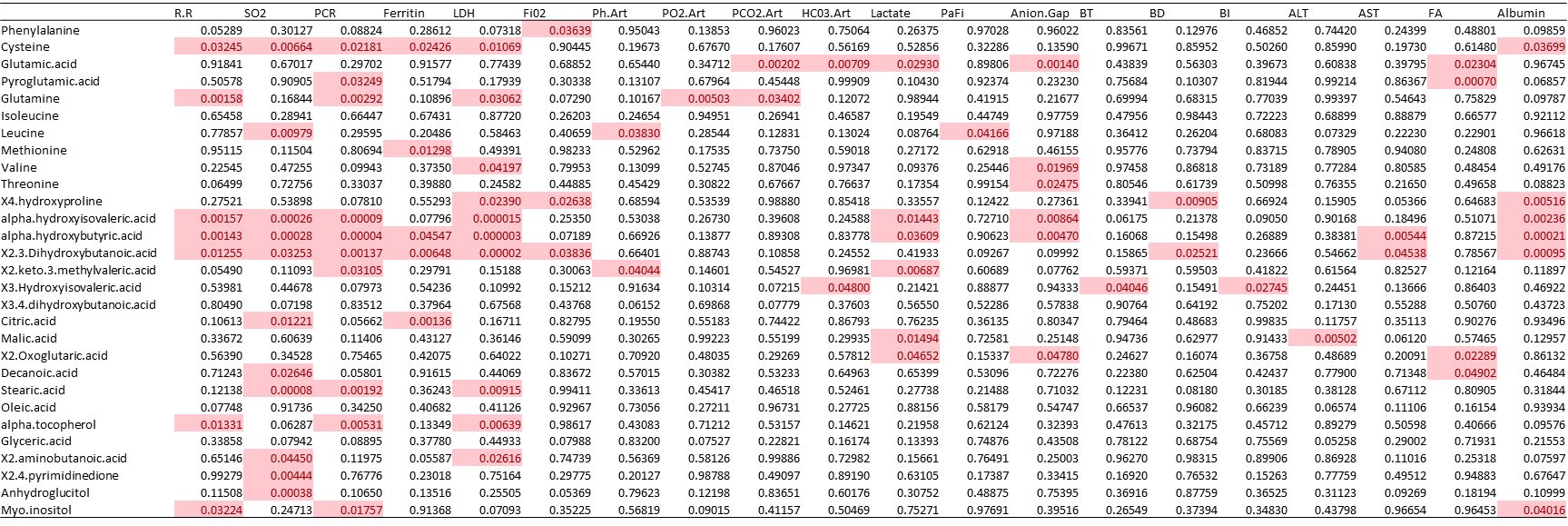


**Supplemental table 2. p-values of Spearman´s correlation analysis in image 3**

**Supplemental table 3. P-values for the generalized linear mixed model analysis adjusted by Obesity and Hypertension, p-value<0.05 means significant effect of patient condition.**

| # | Metabolite | χ^2^ | Df | p-value |
| --- | --- | --- | --- | --- |
| 1 | Phenylalanine | 61.681 | 2 | 4.039×10^-14^ *** |
| 2 | Cysteine | 32.665 | 2 | 8.071x10^-8^*** |
| 3 | Glutamic acid | 51.22 | 2 | 0.0002328 *** |
| 4 | Pyroglutamic acid | 16.73 | 2 | 0.0002328 *** |
| 5 | Glutamine | 20.906 | 2 | 2.886x10^-5^ *** |
| 6 | Isoleucine | 28.967 | 2 | 5.128 x10^-7^ *** |
| 7 | Leucine | 16.102 | 2 | 0.0003187 *** |
| 8 | Valine | 18.048 | 2 | 0.0001205 *** |
| 9 | Threonine | 64.356 | 2 | 1.06x10^-14^ *** |
| 10 | 4 hydroxyproline | 29.919 | 2 | 3.185x10^-7^ *** |
| 11 | α-Hydroxyisovaleric acid | 44.355 | 2 | 2.336x10^-10^ *** |
| 12 | 2,3-Dihydroxybutanoic acid | 22.47 | 2 | 1.32x10^-5^ *** |
| 13 | 2-Keto-3-methylvaleric acid | 15.992 | 2 | 0.0003368 *** |
| 14 | 3-Hydroxyisovaleric.acid | 29.769 | 2 | 3.434x10^-7^ *** |
| 15 | 3,4-Dihydroxybutanoic acid | 7.9481 | 2 | 0.0188 * |
| 16 | Citric acid | 14939595 | 2 | <2.2x10^-16^ *** |
| 17 | Malic acid | 25.307 | 2 | 3.197x10^-6^ *** |
| 18 | α-Ketoglutarate | 22.606 | 2 | 1.23x10^-5^ *** |
| 19 | Decanoic acid | 12.151 | 2 | 0.002299 ** |
| 20 | Stearic acid | 35.178 | 2 | 2.297x10^-8^ *** |
| 21 | Oleic acid | 5.9662 | 2 | 0.05064 |
| 22 | α-tocopherol | 25.421 | 2 | 3.02x10^-6^ *** |
| 23 | 2-Aminobutanoic.acid | 14.043 | 2 | 0.0008925 *** |
| 24 | 2,4-Pyrimidinedione | 22.544 | 2 | 1.273x10^-5^ *** |
| 25 | Anhydroglucitol | 367461 | 2 | < 2.2x10^-16^ *** |
| 26 | Myo-inositol | 12.952 | 2 | 0.00154 ** |

Supplemental table 3. p values for the generalized linear mixed model analysis.

**Supplemental table 4. P-values for the generalized linear model analysis, only for models with Age or BMI effect on Metabolites. Condition: Mild or Severe**

| **Metabolite** | **Factor** | χ^2^ | **Df** | **p-value** |
| --- | --- | --- | --- | --- |
| Anhydroglucitol | Age | 5.15 | 1 | 0.02320 * |
|  | Condition | 10.62 | 2 | 0.00494 ** |
|  | Age*condition | 6.92 | 2 | 0.03131 * |
|  |  |  |  |  |
| 3-Hydroxyisovaleric acid | Age | 3.57 | 1 | 0.05876 |
|  | Condition | 8.67 | 2 | 0.01308 * |
|  | Age*Condition | 6.23 | 2 | 0.04427 * |
|  |  |  |  |  |
| Threonic acid | Age | 8.00 | 1 | 0.004667 ** |
|  | Condition | 8.56 | 2 | 0.013833 * |
|  | Age*Condition | 0.89 | 2 | 0.639387 |
|  |  |  |  |  |
| 3-Hydroxyisovaleric acid | BMI | 6.90 | 1 | 0.008612 ** |
|  | Condition | 20.55 | 2 | 3.445×10^-5^ *** |
|  | BMI*Condition | 6.63 | 2 | 0.036190 * |
| Citric acid | BMI | 6.69 | 1 | 0.009653 ** |
|  | Condition | 40.94 | 2 | 1.287×10^-9^*** |
|  | BMI*Condition | 1.05 | 2 | 0.590071 |

Supplemental table 4. P-values for the generalized linear model analysis, only for models with Age or BMI effect on Metabolites. Condition: Mild or Severe
